# 3-(2-Chloro-3,3,3-trifluoro­prop-1-en-1-yl)-2,2-dimethyl-*N*-[3-(trifluoro­meth­yl)phen­yl]cyclo­propane­carboxamide

**DOI:** 10.1107/S1600536812020922

**Published:** 2012-05-16

**Authors:** Guo-Wu Rao, Xiao-Min Li, Na-Bo Sun

**Affiliations:** a College of Pharmaceutical Science, Zhejiang University of Technology, Hangzhou, 310014, People’s Republic of China; b College of Biology and Environmental Engineering, Zhejiang Shuren University, Hangzhou, 310015, People’s Republic of China

## Abstract

In the title mol­ecule, C_16_H_14_ClF_6_NO, the cyclo­propane ring forms a dihedral angle of 70.82 (18)° with the benzene ring. The torsion angles about the ethyl­ene and amide bonds are −2.2 (5) (Cl—C—C—C) and 0.8 (5)° (O—C—N—C). A supra­molecular chain propagated by glide symmetry along [001] and mediated by N—H⋯O hydrogen bonds is observed in the crystal packing.

## Related literature

For the biological activity of pyrethroids, see: Chen *et al.* (1991 ▶); Sun *et al.* (2007 ▶, 2008 ▶). For the synthesis of the title compound, see: Sun *et al.* (2007 ▶). 
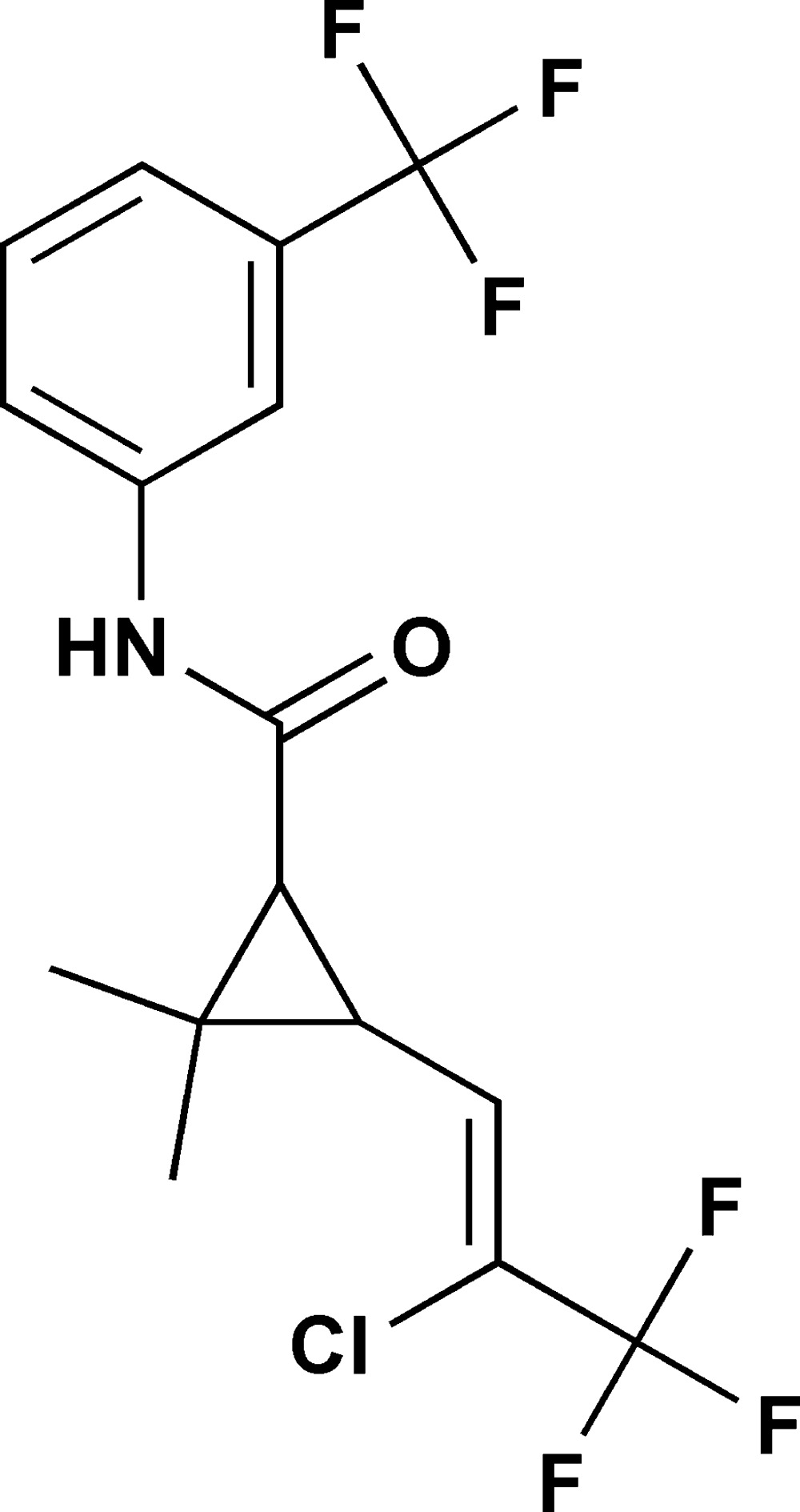


## Experimental

### 

#### Crystal data

C_16_H_14_ClF_6_NO
*M* *_r_* = 385.73
Monoclinic, 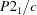

*a* = 11.006 (3) Å
*b* = 16.699 (4) Å
*c* = 9.659 (2) Å
β = 93.009 (3)°
*V* = 1772.8 (7) Å^3^
*Z* = 4
Mo *K*α radiation
μ = 0.28 mm^−1^
*T* = 298 K
0.60 × 0.13 × 0.12 mm

#### Data collection

Bruker SMART CCD diffractometer
Absorption correction: multi-scan (*SADABS*; Bruker, 1997 ▶) *T*_min_ = 0.846, *T*_max_ = 0.967
7354 measured reflections
3120 independent reflections
2247 reflections with *I* > 2σ(*I*)
*R*_int_ = 0.026

#### Refinement

*R*[*F* ^2^ > 2σ(*F* ^2^)] = 0.064
*wR*(*F* ^2^) = 0.175
*S* = 1.04
3120 reflections
256 parameters
H-atom parameters constrained
Δρ_max_ = 0.52 e Å^−3^
Δρ_min_ = −0.48 e Å^−3^

### 

Data collection: *SMART* (Bruker, 1997 ▶); cell refinement: *SAINT* (Bruker, 1997 ▶); data reduction: *SAINT*; program(s) used to solve structure: *SHELXS97* (Sheldrick, 2008 ▶); program(s) used to refine structure: *SHELXL97* (Sheldrick, 2008 ▶); molecular graphics: *ORTEP-3 for Windows* (Farrugia, 1997 ▶); software used to prepare material for publication: *WinGX* (Farrugia, 1999 ▶).

## Supplementary Material

Crystal structure: contains datablock(s) I, global. DOI: 10.1107/S1600536812020922/tk5094sup1.cif

Structure factors: contains datablock(s) I. DOI: 10.1107/S1600536812020922/tk5094Isup2.hkl

Supplementary material file. DOI: 10.1107/S1600536812020922/tk5094Isup3.cdx

Supplementary material file. DOI: 10.1107/S1600536812020922/tk5094Isup4.cml

Additional supplementary materials:  crystallographic information; 3D view; checkCIF report

## Figures and Tables

**Table 1:** Hydrogen-bond geometry (Å, °)

*D*—H⋯*A*	*D*—H	H⋯*A*	*D*⋯*A*	*D*—H⋯*A*
N1—H1⋯O1^i^	0.86	2.15	2.974 (3)	161

Symmetry code: (i) 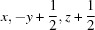.

**Fig. 1.**
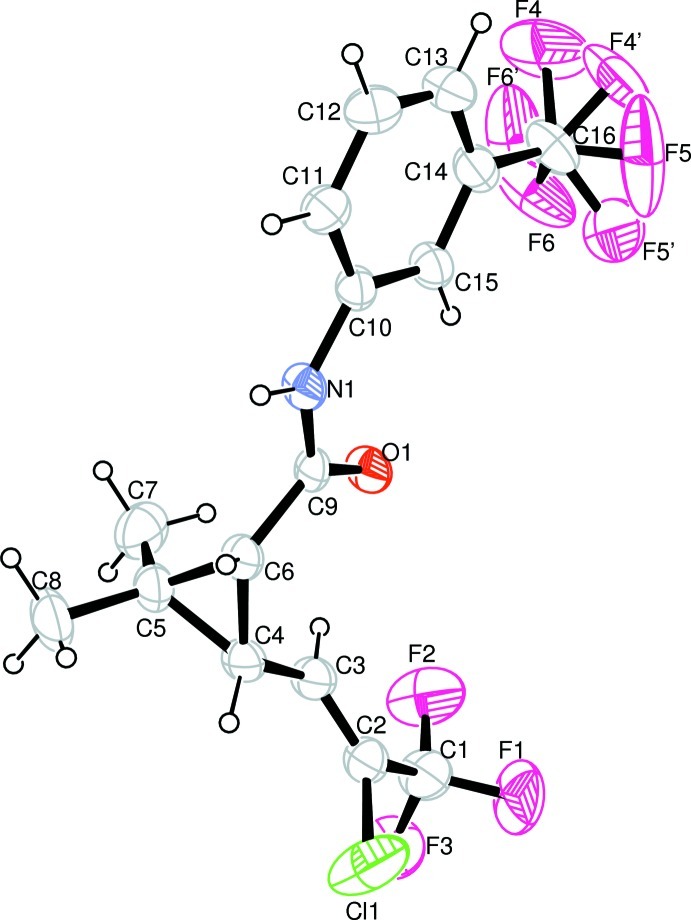
The molecular structure of (I), shown with 30% probability displacement ellipsoids. The F atoms of the CF3 group have 50% occupancy factors.

## supplementary crystallographic information

### Comment

Pyrethroids have a high potential for biological activity with low toxicity and good environmental compatibility. These have been widely used in pesticides (Chen *et al.*, 1991; Sun *et al.*, 2007, 2008). In continuation of our studies of biological activities in pyrethroids, we have obtained a colourless crystalline compound, (I). The structure was confirmed by single-crystal X-ray diffraction.

The molecular structure of (I) is illustrated in Fig. 1. In (I), the cyclopropane ring (C4—C6) forms dihedral angles of 89.70 (23) and 70.82 (18)° with the least-squares planes of the (C5,C7,C8) plane and the phenyl ring, respectively. The Cl1—C2—C3—C4 and O1—C9—N1—C10 torsion angles are -2.2 (5) and 0.8 (5)°, respectively. A supramolecular chain propagated by glide symmetry along [001] and mediated by N—H···O hydrogen bonds, Table 1, is observed in the crystal packing.

### Experimental

The title compound was synthesized according to the literature procedure (Sun *et al.*, 2007). A solution of the compound in ethanol was concentrated gradually at room temperature to afford colourless blocks.

### Refinement

The H-atoms were placed in calculated positions [N—H = 0.86 Å; C—H = 0.93 to 0.98 Å, *U*_iso_(H) = 1.2*U*_eq_(C,N)] and were included in the refinement in the riding model approximation. The CF_3_ group was disordered and modelled over two positions of equal weight.

### Crystal data

**Table:**

C_16_H_14_ClF_6_NO	*Z* = 4
*M**_r_* = 385.73	*F*(000) = 784
Monoclinic, *P*2_1_/*c*	*D*_x_ = 1.445 Mg m^−^^3^
Hall symbol: -P 2ybc	Mo *K*α radiation, λ = 0.71073 Å
*a* = 11.006 (3) Å	µ = 0.28 mm^−^^1^
*b* = 16.699 (4) Å	*T* = 298 K
*c* = 9.659 (2) Å	Block, colourless
β = 93.009 (3)°	0.60 × 0.13 × 0.12 mm
*V* = 1772.8 (7) Å^3^	

### Data collection

**Table:**

Bruker SMART CCD diffractometer	3120 independent reflections
Radiation source: fine-focus sealed tube	2247 reflections with *I* > 2σ(*I*)
Graphite monochromator	*R*_int_ = 0.026
φ and ω scans	θ_max_ = 25.0°, θ_min_ = 1.9°
Absorption correction: multi-scan (*SADABS*; Bruker, 1997)	*h* = −13→12
*T*_min_ = 0.846, *T*_max_ = 0.967	*k* = −19→19
7354 measured reflections	*l* = −10→11

### Refinement

**Table:**

Refinement on *F*^2^	Secondary atom site location: difference Fourier map
Least-squares matrix: full	Hydrogen site location: inferred from neighbouring sites
*R*[*F*^2^ > 2σ(*F*^2^)] = 0.064	H-atom parameters constrained
*wR*(*F*^2^) = 0.175	*w* = 1/[σ^2^(*F*_o_^2^) + (0.0682*P*)^2^ + 1.1818*P*] where *P* = (*F*_o_^2^ + 2*F*_c_^2^)/3
*S* = 1.04	(Δ/σ)_max_ < 0.001
3120 reflections	Δρ_max_ = 0.52 e Å^−^^3^
256 parameters	Δρ_min_ = −0.48 e Å^−^^3^
0 restraints	Extinction correction: *SHELXL97* (Sheldrick, 2008), Fc^*^=kFc[1+0.001xFc^2^λ^3^/sin(2θ)]^-1/4^
Primary atom site location: structure-invariant direct methods	Extinction coefficient: 0.0120 (19)

### Special details

**Table:**

Geometry. All e.s.d.'s (except the e.s.d. in the dihedral angle between two l.s. planes) are estimated using the full covariance matrix. The cell e.s.d.'s are taken into account individually in the estimation of e.s.d.'s in distances, angles and torsion angles; correlations between e.s.d.'s in cell parameters are only used when they are defined by crystal symmetry. An approximate (isotropic) treatment of cell e.s.d.'s is used for estimating e.s.d.'s involving l.s. planes.
Refinement. Refinement of *F*^2^ against ALL reflections. The weighted *R*-factor *wR* and goodness of fit *S* are based on *F*^2^, conventional *R*-factors *R* are based on *F*, with *F* set to zero for negative *F*^2^. The threshold expression of *F*^2^ > σ(*F*^2^) is used only for calculating *R*-factors(gt) *etc*. and is not relevant to the choice of reflections for refinement. *R*-factors based on *F*^2^ are statistically about twice as large as those based on *F*, and *R*- factors based on ALL data will be even larger.

### Fractional atomic coordinates and isotropic or equivalent isotropic displacement parameters (Å^2^)

**Table:**

	*x*	*y*	*z*	*U*_iso_*/*U*_eq_	Occ. (<1)
C1	1.3041 (4)	0.0980 (3)	−0.1232 (4)	0.0913 (12)	
C2	1.3072 (3)	0.1266 (2)	0.0223 (3)	0.0685 (9)	
C3	1.2130 (3)	0.12532 (19)	0.0995 (3)	0.0630 (8)	
H3	1.1393	0.1088	0.0573	0.076*	
C4	1.2130 (3)	0.14749 (19)	0.2459 (3)	0.0619 (8)	
H4	1.2944	0.1552	0.2899	0.074*	
C5	1.1223 (3)	0.1129 (2)	0.3411 (3)	0.0721 (9)	
C8	1.1705 (4)	0.0951 (3)	0.4888 (4)	0.1193 (18)	
H8B	1.2049	0.0423	0.4928	0.179*	
H8A	1.1049	0.0983	0.5503	0.179*	
H8C	1.2319	0.1336	0.5162	0.179*	
C7	1.0243 (4)	0.0577 (2)	0.2842 (5)	0.0998 (13)	
H7A	0.9567	0.0590	0.3432	0.150*	
H7B	1.0556	0.0041	0.2805	0.150*	
H7C	0.9978	0.0747	0.1926	0.150*	
C6	1.1158 (3)	0.20070 (18)	0.3045 (3)	0.0560 (7)	
H6	1.1463	0.2363	0.3792	0.067*	
C9	1.0126 (2)	0.23407 (16)	0.2185 (2)	0.0476 (6)	
C10	0.8386 (3)	0.32746 (16)	0.2473 (3)	0.0527 (7)	
C11	0.7832 (3)	0.37131 (19)	0.3483 (3)	0.0663 (8)	
H11	0.8163	0.3716	0.4389	0.080*	
C12	0.6798 (3)	0.4141 (2)	0.3148 (4)	0.0818 (10)	
H12	0.6429	0.4433	0.3831	0.098*	
C13	0.6299 (3)	0.4144 (2)	0.1813 (4)	0.0798 (10)	
H13	0.5598	0.4437	0.1588	0.096*	
C14	0.6848 (3)	0.37073 (18)	0.0813 (4)	0.0665 (8)	
C15	0.7895 (3)	0.32759 (18)	0.1131 (3)	0.0608 (8)	
H15	0.8267	0.2988	0.0445	0.073*	
C16	0.6311 (4)	0.3696 (3)	−0.0649 (5)	0.0911 (12)	
Cl1	1.44791 (11)	0.15833 (11)	0.08073 (14)	0.1439 (7)	
F1	1.3420 (3)	0.15215 (19)	−0.2109 (3)	0.1418 (12)	
F2	1.1945 (3)	0.0778 (2)	−0.1683 (3)	0.1437 (13)	
F3	1.3764 (3)	0.03595 (16)	−0.1382 (3)	0.1264 (10)	
N1	0.9449 (2)	0.28521 (14)	0.2908 (2)	0.0565 (6)	
H1	0.9703	0.2932	0.3754	0.068*	
O1	0.98962 (18)	0.21704 (13)	0.09703 (18)	0.0643 (6)	
F4	0.5087 (8)	0.3754 (17)	−0.0623 (13)	0.165 (5)	0.50
F5	0.661 (3)	0.4283 (13)	−0.1320 (16)	0.214 (9)	0.50
F6	0.643 (2)	0.3051 (10)	−0.1269 (15)	0.152 (9)	0.50
F4'	0.568 (2)	0.4289 (10)	−0.1007 (11)	0.153 (6)	0.50
F5'	0.7204 (9)	0.3700 (15)	−0.1603 (8)	0.132 (4)	0.50
F6'	0.575 (3)	0.3062 (10)	−0.0982 (18)	0.177 (9)	0.50

### Atomic displacement parameters (Å^2^)

**Table:**

	*U*^11^	*U*^22^	*U*^33^	*U*^12^	*U*^13^	*U*^23^
C1	0.094 (3)	0.121 (3)	0.060 (2)	0.026 (3)	0.017 (2)	−0.008 (2)
C2	0.072 (2)	0.083 (2)	0.0508 (18)	0.0154 (17)	0.0072 (15)	0.0018 (15)
C3	0.0602 (18)	0.081 (2)	0.0475 (16)	0.0132 (15)	0.0012 (14)	−0.0018 (14)
C4	0.0577 (17)	0.086 (2)	0.0414 (15)	0.0140 (15)	−0.0021 (12)	0.0013 (14)
C5	0.084 (2)	0.081 (2)	0.0524 (18)	0.0249 (19)	0.0141 (16)	0.0172 (16)
C8	0.149 (4)	0.148 (4)	0.062 (2)	0.073 (3)	0.023 (2)	0.045 (2)
C7	0.111 (3)	0.072 (2)	0.120 (4)	0.003 (2)	0.037 (3)	0.014 (2)
C6	0.0630 (17)	0.0717 (19)	0.0329 (13)	0.0080 (14)	−0.0013 (12)	−0.0027 (12)
C9	0.0560 (15)	0.0559 (15)	0.0308 (13)	−0.0004 (12)	0.0011 (11)	0.0031 (11)
C10	0.0588 (16)	0.0527 (16)	0.0466 (15)	0.0036 (13)	0.0027 (12)	0.0037 (12)
C11	0.071 (2)	0.073 (2)	0.0561 (18)	0.0065 (16)	0.0095 (15)	−0.0040 (15)
C12	0.079 (2)	0.085 (2)	0.082 (3)	0.0180 (19)	0.020 (2)	−0.0061 (19)
C13	0.067 (2)	0.076 (2)	0.097 (3)	0.0186 (17)	0.0053 (19)	0.010 (2)
C14	0.0655 (19)	0.0607 (18)	0.073 (2)	0.0051 (15)	−0.0035 (16)	0.0097 (16)
C15	0.0709 (19)	0.0603 (17)	0.0510 (17)	0.0113 (15)	0.0013 (14)	0.0012 (14)
C16	0.089 (3)	0.093 (3)	0.088 (3)	0.022 (3)	−0.026 (2)	0.014 (3)
Cl1	0.0863 (8)	0.2456 (18)	0.1022 (9)	−0.0409 (9)	0.0265 (6)	−0.0419 (10)
F1	0.192 (3)	0.173 (3)	0.0640 (15)	0.027 (2)	0.0439 (17)	0.0278 (16)
F2	0.109 (2)	0.251 (4)	0.0706 (15)	0.001 (2)	−0.0003 (14)	−0.0609 (19)
F3	0.152 (2)	0.133 (2)	0.0976 (19)	0.0426 (18)	0.0355 (16)	−0.0290 (15)
N1	0.0674 (15)	0.0694 (15)	0.0320 (11)	0.0117 (12)	−0.0038 (10)	−0.0053 (10)
O1	0.0720 (13)	0.0879 (15)	0.0323 (10)	0.0193 (11)	−0.0052 (9)	−0.0060 (9)
F4	0.088 (5)	0.259 (15)	0.142 (8)	0.039 (7)	−0.058 (5)	−0.037 (9)
F5	0.34 (2)	0.183 (14)	0.107 (10)	−0.117 (14)	−0.092 (13)	0.088 (10)
F6	0.200 (14)	0.154 (14)	0.093 (6)	0.098 (13)	−0.076 (9)	−0.054 (9)
F4'	0.188 (13)	0.145 (10)	0.122 (6)	0.105 (11)	−0.031 (9)	0.024 (7)
F5'	0.116 (5)	0.212 (11)	0.068 (3)	0.017 (7)	−0.009 (3)	0.024 (6)
F6'	0.239 (17)	0.142 (13)	0.139 (10)	−0.088 (13)	−0.094 (11)	0.044 (9)

### Geometric parameters (Å, º)

**Table:**

C1—F2	1.305 (5)	C9—O1	1.221 (3)
C1—F3	1.319 (4)	C9—N1	1.351 (3)
C1—F1	1.321 (5)	C10—C15	1.378 (4)
C1—C2	1.483 (5)	C10—C11	1.387 (4)
C2—C3	1.309 (4)	C10—N1	1.411 (3)
C2—Cl1	1.704 (4)	C11—C12	1.367 (5)
C3—C4	1.462 (4)	C11—H11	0.9300
C3—H3	0.9300	C12—C13	1.376 (5)
C4—C5	1.507 (4)	C12—H12	0.9300
C4—C6	1.522 (4)	C13—C14	1.375 (5)
C4—H4	0.9800	C13—H13	0.9300
C5—C7	1.500 (5)	C14—C15	1.380 (4)
C5—C6	1.509 (4)	C14—C16	1.502 (5)
C5—C8	1.525 (5)	C15—H15	0.9300
C8—H8B	0.9600	C16—F5	1.230 (10)
C8—H8A	0.9600	C16—F6	1.242 (11)
C8—H8C	0.9600	C16—F4'	1.246 (9)
C7—H7A	0.9600	C16—F6'	1.258 (12)
C7—H7B	0.9600	C16—F4	1.352 (10)
C7—H7C	0.9600	C16—F5'	1.381 (10)
C6—C9	1.481 (4)	N1—H1	0.8600
C6—H6	0.9800		
			
F2—C1—F3	108.2 (4)	N1—C9—C6	112.0 (2)
F2—C1—F1	106.3 (4)	C15—C10—C11	119.7 (3)
F3—C1—F1	104.8 (3)	C15—C10—N1	123.9 (3)
F2—C1—C2	112.0 (3)	C11—C10—N1	116.3 (3)
F3—C1—C2	111.9 (3)	C12—C11—C10	120.0 (3)
F1—C1—C2	113.3 (4)	C12—C11—H11	120.0
C3—C2—C1	123.7 (3)	C10—C11—H11	120.0
C3—C2—Cl1	123.3 (3)	C11—C12—C13	120.7 (3)
C1—C2—Cl1	112.9 (3)	C11—C12—H12	119.7
C2—C3—C4	126.0 (3)	C13—C12—H12	119.7
C2—C3—H3	117.0	C14—C13—C12	119.2 (3)
C4—C3—H3	117.0	C14—C13—H13	120.4
C3—C4—C5	121.8 (3)	C12—C13—H13	120.4
C3—C4—C6	122.9 (2)	C13—C14—C15	120.9 (3)
C5—C4—C6	59.8 (2)	C13—C14—C16	120.1 (3)
C3—C4—H4	114.0	C15—C14—C16	119.0 (3)
C5—C4—H4	114.0	C10—C15—C14	119.4 (3)
C6—C4—H4	114.0	C10—C15—H15	120.3
C7—C5—C4	119.9 (3)	C14—C15—H15	120.3
C7—C5—C6	119.1 (3)	F5—C16—F6	113.5 (12)
C4—C5—C6	60.6 (2)	F5—C16—F4'	51.8 (11)
C7—C5—C8	115.5 (4)	F6—C16—F4'	128.8 (9)
C4—C5—C8	115.9 (3)	F5—C16—F6'	132.7 (8)
C6—C5—C8	114.7 (3)	F6—C16—F6'	37.9 (10)
C5—C8—H8B	109.5	F4'—C16—F6'	110.0 (9)
C5—C8—H8A	109.5	F5—C16—F4	104.5 (11)
H8B—C8—H8A	109.5	F6—C16—F4	101.7 (9)
C5—C8—H8C	109.5	F4'—C16—F4	53.9 (6)
H8B—C8—H8C	109.5	F6'—C16—F4	65.7 (9)
H8A—C8—H8C	109.5	F5—C16—F5'	54.8 (12)
C5—C7—H7A	109.5	F6—C16—F5'	65.5 (8)
C5—C7—H7B	109.5	F4'—C16—F5'	102.4 (8)
H7A—C7—H7B	109.5	F6'—C16—F5'	101.0 (10)
C5—C7—H7C	109.5	F4—C16—F5'	139.2 (7)
H7A—C7—H7C	109.5	F5—C16—C14	112.4 (6)
H7B—C7—H7C	109.5	F6—C16—C14	114.7 (6)
C9—C6—C5	121.5 (3)	F4'—C16—C14	115.8 (7)
C9—C6—C4	123.0 (2)	F6'—C16—C14	114.4 (7)
C5—C6—C4	59.6 (2)	F4—C16—C14	108.9 (6)
C9—C6—H6	114.1	F5'—C16—C14	111.6 (5)
C5—C6—H6	114.1	C9—N1—C10	129.3 (2)
C4—C6—H6	114.1	C9—N1—H1	115.4
O1—C9—N1	123.5 (2)	C10—N1—H1	115.4
O1—C9—C6	124.4 (2)		
			
F2—C1—C2—C3	5.3 (6)	C15—C10—C11—C12	0.4 (5)
F3—C1—C2—C3	−116.4 (4)	N1—C10—C11—C12	179.9 (3)
F1—C1—C2—C3	125.5 (4)	C10—C11—C12—C13	−0.2 (5)
F2—C1—C2—Cl1	−176.8 (3)	C11—C12—C13—C14	0.3 (6)
F3—C1—C2—Cl1	61.6 (4)	C12—C13—C14—C15	−0.7 (5)
F1—C1—C2—Cl1	−56.6 (4)	C12—C13—C14—C16	179.4 (4)
C1—C2—C3—C4	175.5 (3)	C11—C10—C15—C14	−0.7 (4)
Cl1—C2—C3—C4	−2.2 (5)	N1—C10—C15—C14	179.8 (3)
C2—C3—C4—C5	−153.3 (3)	C13—C14—C15—C10	0.9 (5)
C2—C3—C4—C6	134.5 (4)	C16—C14—C15—C10	−179.2 (3)
C3—C4—C5—C7	−3.5 (5)	C13—C14—C16—F5	83 (2)
C6—C4—C5—C7	108.7 (3)	C15—C14—C16—F5	−97 (2)
C3—C4—C5—C6	−112.3 (3)	C13—C14—C16—F6	−145.8 (15)
C3—C4—C5—C8	142.7 (3)	C15—C14—C16—F6	34.3 (16)
C6—C4—C5—C8	−105.0 (4)	C13—C14—C16—F4'	25.6 (15)
C7—C5—C6—C9	2.4 (4)	C15—C14—C16—F4'	−154.3 (14)
C4—C5—C6—C9	112.4 (3)	C13—C14—C16—F6'	−103.9 (18)
C8—C5—C6—C9	−140.6 (3)	C15—C14—C16—F6'	76.2 (18)
C7—C5—C6—C4	−110.0 (3)	C13—C14—C16—F4	−32.7 (14)
C8—C5—C6—C4	107.0 (3)	C15—C14—C16—F4	147.4 (14)
C3—C4—C6—C9	0.5 (5)	C13—C14—C16—F5'	142.2 (12)
C5—C4—C6—C9	−109.9 (3)	C15—C14—C16—F5'	−37.7 (13)
C3—C4—C6—C5	110.4 (4)	O1—C9—N1—C10	0.8 (5)
C5—C6—C9—O1	−65.5 (4)	C6—C9—N1—C10	−178.4 (3)
C4—C6—C9—O1	6.5 (5)	C15—C10—N1—C9	−5.4 (5)
C5—C6—C9—N1	113.7 (3)	C11—C10—N1—C9	175.1 (3)
C4—C6—C9—N1	−174.3 (3)		

### Hydrogen-bond geometry (Å, º)

**Table:**

*D*—H···*A*	*D*—H	H···*A*	*D*···*A*	*D*—H···*A*
N1—H1···O1^i^	0.86	2.15	2.974 (3)	161

Symmetry code: (i) *x*, −*y*+1/2, *z*+1/2.
